# Supplementary figures and images for: Identification and analysis of sucrose synthase gene family associated with polysaccharide biosynthesis in Dendrobium catenatum by transcriptomic analysis
Source: PeerJ. 2022 Apr 5;10:e13222. doi: 10.7717/peerj.13222 (PMC8992646; doi:10.7717/peerj.13222)

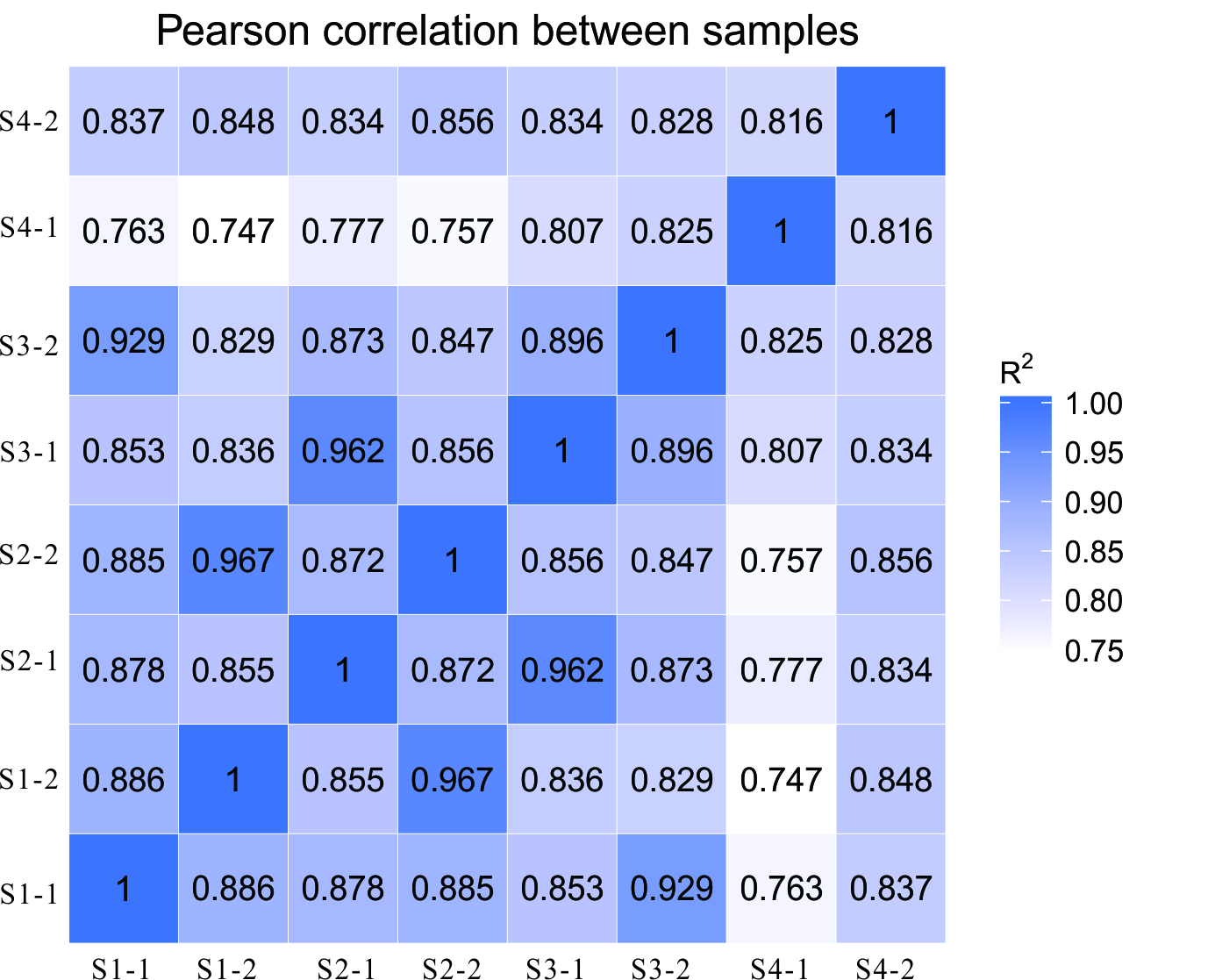

Supplement: Figure S2 [file peerj-10-13222-s002.tif]

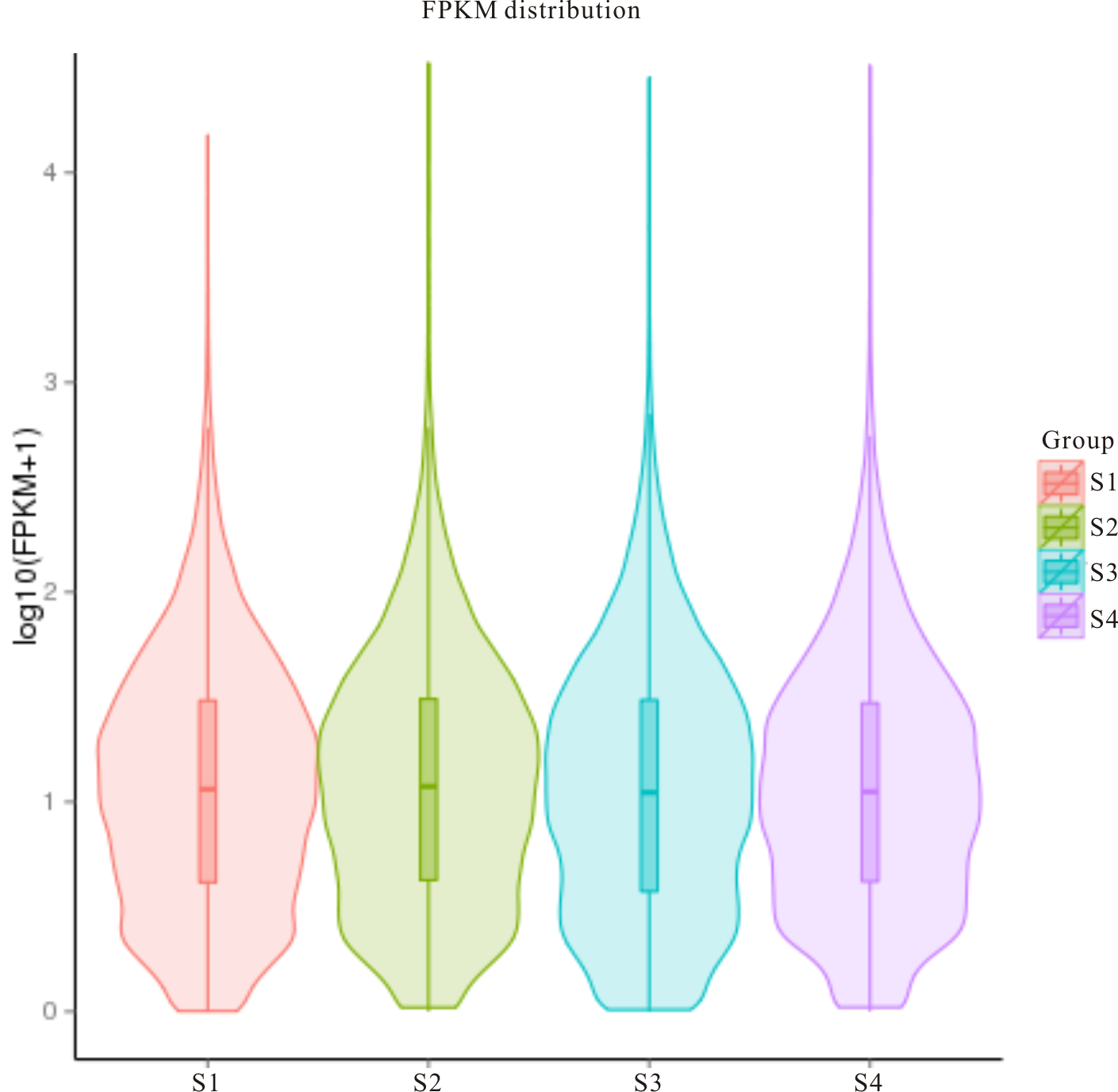

Supplement: Figure S3 [file peerj-10-13222-s003.tif]

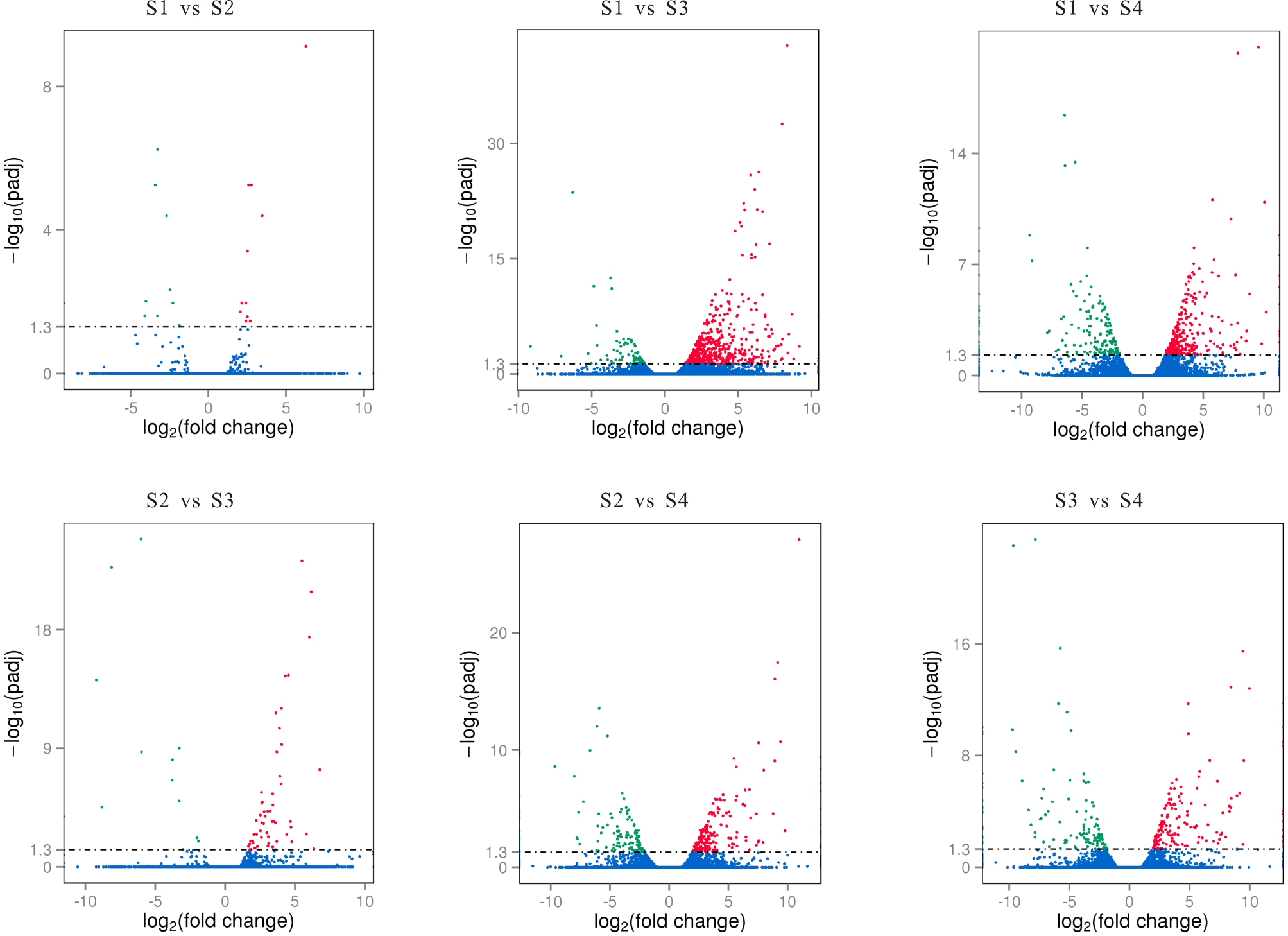

Supplement: Figure S4 [file peerj-10-13222-s004.tif]

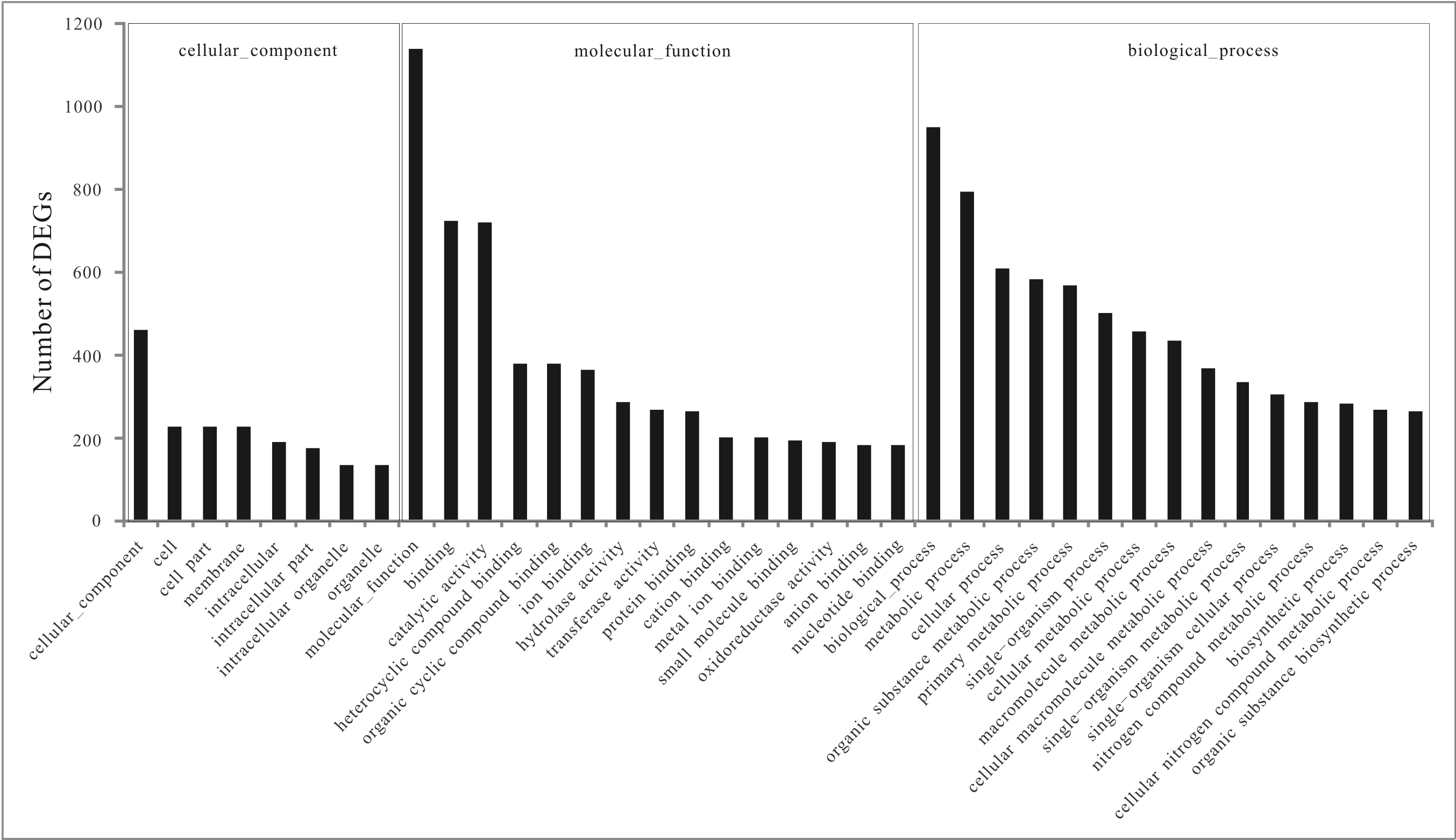

Supplement: Figure S5 — The results are summarized in mainly three categories: biological process, cellular component and molecular function. [file peerj-10-13222-s005.tif]
