# Supplementary material for: Identification and analysis of sucrose synthase gene family associated with polysaccharide biosynthesis in Dendrobium catenatum by transcriptomic analysis
Source: PeerJ. 2022 Apr 5;10:e13222. doi: 10.7717/peerj.13222 (PMC8992646; doi:10.7717/peerj.13222)
Supplement: Table S1 [file peerj-10-13222-s006.docx]

**Table S1. The list of qRT-PCR primers of genes selected in *Dendrobium catenatum*.**

| Gene name | Gene model | Forward Primer | Reverse Primer |
| --- | --- | --- | --- |
| 18S rRNA | Dendrobium_GLEAN_10067105 | CCAGGTCCAGACATAGTAAG | GTACAAAGGGCAGGGACGTA |
| PMM | Dendrobium_GLEAN_10056672 | TTGACCGCATCGCACCTA | CTCGGTCGCAGATTTGTT |
| MPI | Dendrobium_GLEAN_10071332 | GGTAAAGCGACCATTCAGG | CAATCGTCTGCTCAGAGT |
| MPI | Dendrobium_GLEAN_10071336 | GCATGGCGACATCAGATAA | ACGCAAGATTTCAGGAAGG |
|  | Dendrobium_GLEAN_10031143 | TTGTTCCAGGGCATGAGAT | CAAGAACGGCAAGCACCTA |
|  | Dendrobium_GLEAN_10058899 | TACTGTGGCATTTGCCATTC | TGACGACGCCAACAATTTC |
|  | Dendrobium_GLEAN_10059177 | ATCTGCGGCCATCGTACTA | TCTCGGAGGAGGTTCTGAC |
|  | Dendrobium_GLEAN_10118528 | CAATGTGCTGTCCACTTCAGA | AGAAGATGGCTGATTCAATCC |
| srlD | Dendrobium_GLEAN_10023268 | TGGAACTCAGATCACGGTC | AGCTACAGGAAAGTGCACAA |
| srlD | Dendrobium_GLEAN_10023269 | GGAGAGACTGCAGGAGAA | GGCAGGTTGATTGGTACG |
| ALDO | Dendrobium_GLEAN_10023929 | TCCAATGCTACTTGTGGGAAAC | CAGACCAGGAGCTGAGACTA |
| ALDO | Dendrobium_GLEAN_10046247 | GCCCTTCGTCGAAGTTCTA | GATCATCGAGTCCTTGCG |
| ALDO | Dendrobium_GLEAN_10040110 | AGACAATGGACTTGTTCCG | AGAACACCTCAGCCCAGA |
| fruK | Dendrobium_GLEAN_10138390 | GCTCCAAGATAATCCGGCA | ATGTGTACCTCTAGGGCTG |
| fruK | Dendrobium_GLEAN_10024822 | GAGGTCCTGACATTGAGGAA | CATTGCATTTGCACCAGC |
| GMPP | Dendrobium_GLEAN_10109385 | ACAAGCTAATTCTTGCTGCTC | GGTTCCACATCTTCCACAATAA |
| GMPP | Dendrobium_GLEAN_10054900 | TCTTCTAGCTGGAAGCAAGG | AGAAATGGAGACATTTGGACC |
| PFKFB | Dendrobium_GLEAN_10111616 | AACCTGTAGAAGAAGGTTGC | AATCCGACCAGGAAGATATCCA |
| PFKFB | Dendrobium_GLEAN_10121160 | AGAGAGTGGTTCAACAACG | CTGCCAGATTAGCTGCAT |
| GFPT | Dendrobium_GLEAN_10054184 | AAGAGATTCACGAGCAACC | ATATAATTCTCCTGCTGTGCC |
| GNPNAT | Dendrobium_GLEAN_10039040 | GAACATGCTAGGGTTGCAG | TGGAAGTACATCGCCATTTG |
| PGM | Dendrobium_GLEAN_10051178 | AGAGACACTAGACCTACTGG | ATGAAGTTGTGGAGTGGTTAC |
| UAP | Dendrobium_GLEAN_10021051 | CTTGCTGCTCAATCAGCG | CGACTTTCAAAGAACTTGCGA |
| UAP | Dendrobium_GLEAN_10021052 | GCACTCTTCGAGGCATTG | CAACGACTATGAAGGCGGA |
|  | Dendrobium_GLEAN_10126101 | AATGCCATAGTATGCAGTGT | GAAACCCTTGGTGACCATTC |
| AXS | Dendrobium_GLEAN_10045157 | TAGTGGAGAACACTCCTGATGA | GCAAACCATCTCTTAGAACGAC |
| AXS | Dendrobium_GLEAN_10051720 | AGAATGGACTTTATTCCTGGAG | AAGCTTCAAAGGTTCACGAC |
| UXE | Dendrobium_GLEAN_10125412 | AACGGTGAAGGAGTTTGTG | GTAAACTTCAGCATAGTCGCC |
| ASD | Dendrobium_GLEAN_10028711 | GGGCATGGGACATGGATTTA | TTAGCCACTCTCCCTCAACA |
| ASD | Dendrobium_GLEAN_10026326 | AATCTGGCATTCGATGTCC | ATGCTTCTTTGGCGAGTTA |
| ARA | Dendrobium_GLEAN_10067468 | TGGATTCTGGTGCTGTTCA | CCACTCAACCTCAGTTGC |
| ARA | Dendrobium_GLEAN_10066923 | GTTACCTTCTGGTTGGCTG | TGAAGCATAAGGGCACGAG |
| GAE | Dendrobium_GLEAN_10031397 | GACTTCACCTACATTGACGAC | GGTGTTACCAAGGTTGAAGAC |
| GAE | Dendrobium_GLEAN_10128035 | AGGAGATCACTCACACCTACAA | AAGGAGAAATAGGCCATGTCA |
| GALE | Dendrobium_GLEAN_10077593 | AGCAATAATTTGATCGGGACAT | CAGGTTGACCATAAACAGTAGT |
| GALE | Dendrobium_GLEAN_10059583 | CAACAACAATTTGATCGGCAC | TTCAGGTTGCCCATAAACAG |
| UGP | Dendrobium_GLEAN_10053350 | TAATGTTGTTGCTGCAACTGA | CCAAAGTTCCTTTGACACG |
| UGP | Dendrobium_GLEAN_10052658 | AAATGGAGATTATCCCAAACCC | CGTGGAACATTTACTCCGATAG |
| UDGH | Dendrobium_GLEAN_10005633 | ACCTAAGTTCTTGAACGCC | GGCAACTTCTGGAAGACC |
| UDGH | Dendrobium_GLEAN_10048133 | AATGCGTGGAACAGTGAC | TCTCTACCTCGGTGCTGA |
| PGM | Dendrobium_GLEAN_10079430 | TATGCCAACACGAAGACAATC | CCACATCATAACCTCCTTCAG |
| PGM | Dendrobium_GLEAN_10069896 | TGGTACAGGATCAGATCACAT | GCTACTGATACCAACTTCTCG |
|  | Novel00587 | CCAGGTCCAGACATAGTAAG | GTACAAAGGGCAGGGACGTA |
| TPI | Novel04679 | GGAAATTCTTCGTCGGCG | ACCTCAACAACATCCTGTG |
| UXE | Novel01395 | ACATCCTTGAACATCCACAC | CTCTCCTGTAAATGCTGAAACC |
