# Supplementary material for: Identification and analysis of sucrose synthase gene family associated with polysaccharide biosynthesis in Dendrobium catenatum by transcriptomic analysis
Source: PeerJ. 2022 Apr 5;10:e13222. doi: 10.7717/peerj.13222 (PMC8992646; doi:10.7717/peerj.13222)
Supplement: Table S2 [file peerj-10-13222-s007.docx]

**Table S2. The list of qRT-PCR primers of SUS genes in *Dendrobium catenatum*.**

| Gene name | Gene model | Forward Primer | Reverse Primer |
| --- | --- | --- | --- |
| GAPDH | KP719976 | ACCGTGAGGATTGAGAAGGC | TGGCAATGTGGCGGATAAGA |
| DcSUS1 | Dendrobium_GLEAN_10013680 | ACAGGCAGTTGGGTGTTGTT | GCAACGCTCGAACTTGATCC |
| DcSUS2 | Dendrobium_GLEAN_10032668 | GTGCAGCCTGCCTTCTATGA | CAATGTGGAAGCCCGAGACT |
| DcSUS3 | Dendrobium_GLEAN_10069518 | ACCAGAGGCATATTTCCTGCTT | AGAGTTGTAGCCTGCTGTGG |
| DcSUS4 | Dendrobium_GLEAN_10069521 | CGCCTTAGATGACTCGGCTATT | GCACTTCCAAACCAGCACCT |
| DcSUS5 | Dendrobium_GLEAN_10069526 | GTGGAGGAATGCATCGGACT | TAGTGGCCAACATTCGGCAT |
| DcSUS6 | Dendrobium_GLEAN_10089179 | TCCTTTCCGAACCGAGAACAG | AAGCTCTCCGACCAACTTGT |
| DcSUS7 | Dendrobium_GLEAN_10095005 | GCAATGGAACAGTTGCGTGT | TCTGACCTCTTTGCACGGTC |
| DcSUS8 | Dendrobium_GLEAN_10105018 | GCTCTACCGCGTAGTTCACG | TGTGAGCCTTTTGCTCTCCT |
| DcSUS9 | Dendrobium_GLEAN_10115731 | TCTCCATGGCTAGGCTCGAT | TCATAGAACGCAGGCTGAACA |
| DcSUS10 | Dendrobium_GLEAN_10133317 | AGCATTTGGGCTCACAGTCA | TGACTGCATCATTCTTACAAGTCC |
| DcSUS11 | Dendrobium_GLEAN_10114912 | ACGGGGTCCTATGGATCAGT | ATGGCTTCAAAGGTTGCCCT |
| DcSUS12 | Dendrobium_GLEAN_10075975 | ACATTCGCCATAAACGCTGC | GCCCGTTCATTCCTGTCTGA |
| DcSUS13 | Dendrobium_GLEAN_10069519 | AGGAAGCCACACTCACCAAG | TAAACCCCAGCAAGCGTCAT |
| DcSUS14 | Dendrobium_GLEAN_10105017 | CAACTCGCCCGAGAGCTATT | TAGAGGGCAGGTCCTGATGT |
| DcSUS15 | Dendrobium_GLEAN_10063746 | ATGGCTTCTGCCAAGCATCT | TGCACATCACTGACTGCCAT |
