# Supplementary material for: Identification and analysis of sucrose synthase gene family associated with polysaccharide biosynthesis in Dendrobium catenatum by transcriptomic analysis
Source: PeerJ. 2022 Apr 5;10:e13222. doi: 10.7717/peerj.13222 (PMC8992646; doi:10.7717/peerj.13222)
Supplement: Table S4 [file peerj-10-13222-s009.docx]

| FPKM Interval | S1-1 | S1-2 | S2-1 | S2-2 | S3-1 | S3-2 | S4-1 | S4-2 |  |
| --- | --- | --- | --- | --- | --- | --- | --- | --- | --- |
| 0~1 | 15499(37.37%) | 14918(35.97%) | 15240(36.75%) | 14864(35.84%) | 15564(37.53%) | 15867(38.26%) | 15940(38.44%) | 14447(34.84%) | |
| 1~3 | 4165(10.04%) | 4145(9.99%) | 4194(10.11%) | 4266(10.29%) | 4232(10.20%) | 4186(10.09%) | 4378(10.56%) | 4380(10.56%) | |
| 3~15 | 9869(23.80%) | 10007(24.13%) | 9676(23.33%) | 9971(24.04%) | 9567(23.07%) | 9491(22.89%) | 9717(23.43%) | 10486(25.28%) | |
| 15~60 | 8424(20.31%) | 8809(21.24%) | 8661(20.88%) | 8810(21.24%) | 8400(20.25%) | 8337(20.10%) | 7973(19.23%) | 8615(20.77%) | |
| >60 | 3515(8.48%) | 3593(8.66%) | 3701(8.92%) | 3561(8.59%) | 3709(8.94%) | 3591(8.66%) | 3464(8.35%) | 3544(8.55%) | |

**Table S4. The statistic results of FPKM Interval about 8 samples in the study.**
