# Supplementary material for: Identification and analysis of sucrose synthase gene family associated with polysaccharide biosynthesis in Dendrobium catenatum by transcriptomic analysis
Source: PeerJ. 2022 Apr 5;10:e13222. doi: 10.7717/peerj.13222 (PMC8992646; doi:10.7717/peerj.13222)
Supplement: Table S9 [file peerj-10-13222-s014.docx]

| EC ID | Definition | Name |
| --- | --- | --- |
| EC:1.1.1.22 | UDP glucose 6-dehydrogenase | UGDH |
| EC:5.1.3.6 | UDP-glucuronate 4-epimerase | GAE |
| EC:2.4.1.43 | alpha-1,4-galacturonosyltransferase | GAUT |
| EC:3.1.1.11 | pectin methylesterase | PME |
| EC:4.1.1.35 | UDP-glucuronate decarboxylase | UXS1 |
| EC:5.1.3.5 | UDP-arabinose 4-epimerase | UXE |
| EC:2.4.2.24 | 1,4-beta-D-xylan synthase | xysB |
| EC:3.2.1.37 | xylan 1,4-beta-xylosidase | xynB |
| EC:2.4.1.15 | trehalose 6-phosphate synthase | TPS |
| EC:3.1.3.12 | trehalose 6-phosphate phosphatase | TPP |
| EC:3.2.1.122 | maltose-6'-phosphate glucosidase | glvA |
| EC:3.2.1.93 | trehalose-6-phosphate hydrolase | treC |
| EC:3.1.3.90 | maltose 6'-phosphate phosphatase | mapP |
| EC:2.4.1.13 | sucrose synthase | SUS |
| EC:3.2.1.20 | maltase-glucoamylase | MGAM |
| EC:3.2.1.26 | beta-fructofuranosidase | INV |
| EC:3.2.1.48 | sucrase-isomaltas | SI |
| EC:5.3.1.8 | mannose-6-phosphate isomerase | MPI |
| EC:2.7.1.191 | PTS system, mannose-specific IIA component | PTS-Man-EIIA |
| EC:2.7.1.1 | hexokinase | HK |
| EC:3.2.1.21 | beta-glucosidase | BGLU |
| EC:2.4.1.35 | UDP-glucose glucosyltransferase | UGG |
| EC:2.7.1.7 | mannokinase | MK |
| AXS | UDP-apiose/xylose synthase | AXS |
| EC:5.4.99.16 | maltose alpha-D-glucosyltransferase | treS |
| EC:2.4.1.25 | glycogen debranching enzyme | AGL |
| E2.7.1.4 | fructokinase | scrK |
| EC:2.7.1.2 | glucokinase | glk |
| EC:2.7.1.63 | polyphosphate glucokinase | ppgK |
| EC:5.3.1.9 | glucose-6-phosphate isomerase | GPI |

Table S9. The list of full names about enzymes by EC IDs as mentioned in Fig. 3.
