# Supplementary material for: Identification and analysis of sucrose synthase gene family associated with polysaccharide biosynthesis in Dendrobium catenatum by transcriptomic analysis
Source: PeerJ. 2022 Apr 5;10:e13222. doi: 10.7717/peerj.13222 (PMC8992646; doi:10.7717/peerj.13222)
Supplement: Table S10 [file peerj-10-13222-s015.docx]

**Table S10.** The lists of *SUS* gene family in 16 plant species.

| Gene name | Gene_id | Strand | ORF | No. of a.a | No. of introns | 5’-3’ Coordinate | pI | Mw(kD) |
| --- | --- | --- | --- | --- | --- | --- | --- | --- |
| *Arabidopsis thaliana* | | | | | | | | |
| AtSUS1 | AT5G20830 | - | 2424 | 807 | 12 | Chr5 : 7050599-7054032 | 5.83 | 93.00 |
| AtSUS2 | AT5G49190 | - | 2421 | 806 | 11 | Chr5 : 19943369-19947189 | 5.70 | 62.06 |
| AtSUS3 | AT4G02280 |  | 2427 | 808 | 11 | Chr4 : 995166-998719 | 5.85 | 92.00 |
| AtSUS4 | AT3G43190 | - | 2424 | 807 | 11 | Chr3 : 15179204-15182577 | 6.12 | 93.00 |
| AtSUS5 | AT5G37180 | + | 2511 | 836 | 12 | Chr5 : 14718238-14722913 | 6.13 | 95.03 |
| AtSUS6 | AT1G73370 | - | 2826 | 941 | 11 | Chr1 : 27584533-27588326 | 8.13 | 106.88 |
| *Amborella trichopoda* | | | | | | | | |
| AtrSUS1 | ATR0317G021 | - | 2430 | 809 | 14 | scaffold00106:80681-87957 | 6.14 | 92.52 |
| AtrSUS2 | ATR0807G093 | - | 2556 | 851 | 16 | scaffold00044:420024-427185 | 8.35 | 96.71 |
| *Chlamydomonas reinhardtii* | | | | | | | | |
| CrSUS1 | Cre12.g524000 | + | 6945 | 2314 | 23 | chromosome_12:4753113-4765694 | 5.99 | 226.02 |
| *Brachypodium distachyon* | | | | | | | | |
| BdSUS1 | Bradi1g20890 | + | 2451 | 816 | 14 | 16789196-16793519 | 6.10 | 93.16 |
| BdSUS2 | Bradi1g29570 | + | 2592 | 863 | 16 | 25158373-25162521 | 8.23 | 98.93 |
| BdSUS3 | Bradi1g46670 | + | 2424 | 807 | 14 | 45377885-45382275 | 6.01 | 92.13 |
| BdSUS4 | Bradi1g60320 | + | 2445 | 814 | 13 | 59850702-59855241 | 5.87 | 92.54 |
| BdSUS5 | Bradi1g62957 | + | 2427 | 808 | 14 | 62379261-62385942 | 6.05 | 92.31 |
| BdSUS6 | Bradi3g60687 | + | 2799 | 932 | 15 | 59321061-59325376 | 7.18 | 106.00 |
| *Brassica rapa* | | | | | | | | |
| BrSUS1 | Brara.C00954 | - | 2421 | 806 | 10 | A03 : 4450794-4454625 | 5.76 | 92.48 |
| BrSUS2 | Brara.E01375 | - | 2541 | 846 | 12 | A05 : 8601273-8605807 | 6.18 | 96.21 |
| BrSUS3 | Brara.F03015 | + | 2397 | 798 | 13 | A06 : 24153728-24157644 | 6.09 | 91.06 |
| BrSUS4 | Brara.G02318 | + | 1227 | 408 | 5 | A07 : 19835972-19839793 | 8.55 | 45.99 |
| BrSUS5 | Brara.G02800 | + | 1572 | 523 | 5 | A07 : 22543825-22545785 | 6.87 | 59.48 |
| BrSUS6 | Brara.G03267 | + | 1230 | 409 | 5 | A07 : 25091028-25094696 | 8.70 | 46.79 |
| BrSUS7 | Brara.I00137 | + | 2436 | 811 | 11 | A09 : 786483-790395 | 5.83 | 92.34 |
| BrSUS8 | Brara.J01546 | + | 2418 | 805 | 11 | A10 : 13465780-13469161 | 5.80 | 92.21 |
| *Dendrobium catenatum* | | | | | | | | |
| DcSUS1 | Dendrobium_GLEAN_10013680 | + | 891 | 296 | 0 | scaffold18586:1848-5163 | 6.49 | 33.46 |
| DcSUS2 | Dendrobium_GLEAN_10032668 | + | 2418 | 805 | 13 | scaffold10161:31952-36549 | 6.02 | 91.65 |
| DcSUS3 | Dendrobium_GLEAN_10069518 | + | 825 | 274 | 1 | scaffold4378:1138-2257 | 8.98 | 31.60 |
| DcSUS4 | Dendrobium_GLEAN_10069521 | - | 1251 | 416 | 6 | scaffold4378:18072-30379 | 6.09 | 46.99 |
| DcSUS5 | Dendrobium_GLEAN_10069526 | - | 861 | 286 | 5 | scaffold4378:50731-54138 | 9.05 | 32.64 |
| DcSUS6 | Dendrobium_GLEAN_10089179 | - | 1617 | 538 | 0 | scaffold2581:4778-6391 | 6.21 | 59.91 |
| DcSUS7 | Dendrobium_GLEAN_10095005 | + | 1197 | 398 | 0 | scaffold2157:110070-111263 | 7.62 | 44.65 |
| DcSUS8 | Dendrobium_GLEAN_10105018 | - | 285 | 94 | 1 | scaffold1516:154975-155351 | 4.72 | 10.53 |
| DcSUS9 | Dendrobium_GLEAN_10115731 | + | 2454 | 817 | 0 | scaffold939:12633-16921 | 6.01 | 92.95 |
| DcSUS10 | Dendrobium_GLEAN_10133317 | - | 2466 | 821 | 0 | scaffold263:133585-138259 | 6.76 | 92.66 |
| DcSUS11 | Dendrobium_GLEAN_10114912 | - | 1950 | 649 | 8 | scaffold986:138945-175168 | 5.91 | 72.73 |
| DcSUS12 | Dendrobium_GLEAN_10075975 | + | 2838 | 945 | 0 | scaffold3686:88092-90929 | 5.63 | 105.38 |
| DcSUS13 | Dendrobium_GLEAN_10069519 | - | 309 | 102 | 0 | scaffold4378:6958-8971 | 9.91 | 11.95 |
| DcSUS14 | Dendrobium_GLEAN_10105017 | - | 870 | 289 | 0 | scaffold1516:152719-154872 | 8.92 | 33.31 |
| DcSUS15 | Dendrobium_GLEAN_10063746 | + | 951 | 316 | 0 | scaffold5068:67096-68046 | 5.14 | 34.59 |
| *Glycine max* | | | | | | | | |
| GmSUS1 | Glyma.02G240400 | + | 2523 | 840 | 14 | 42892859-42897937 | 6.81 | 95.33 |
| GmSUS2 | Glyma.03G216300 | - | 2439 | 812 | 14 | 42038409-42044019 | 5.76 | 92.31 |
| GmSUS3 | Glyma.09G073600 | - | 2433 | 810 | 11 | 7810856-7815282 | 6.02 | 93.66 |
| GmSUS4 | Glyma.09G167000 | - | 2766 | 921 | 14 | 39104025-39109505 | 6.61 | 104.15 |
| GmSUS5 | Glyma.11G212700 | - | 2538 | 845 | 14 | 30547491-30552238 | 6.53 | 95.77 |
| GmSUS6 | Glyma.13G114000 | - | 2418 | 805 | 11 | 22767977-22771945 | 6.04 | 92.24 |
| GmSUS7 | Glyma.14G209900 | + | 2523 | 840 | 14 | 47516076-47521414 | 7.02 | 95.28 |
| GmSUS8 | Glyma.15G151000 | - | 2409 | 802 | 14 | 12497421-12504514 | 5.84 | 91.57 |
| GmSUS9 | Glyma.15G182600 | - | 2421 | 806 | 12 | 17910482-17915416 | 5.87 | 92.72 |
| GmSUS10 | Glyma.16G217200 | - | 2763 | 920 | 14 | 37414392-37419628 | 6.66 | 103.91 |
| GmSUS11 | Glyma.17G045800 | + | 2418 | 805 | 11 | 3406111-3410110 | 5.93 | 92.19 |
| GmSUS12 | Glyma.19G212800 | - | 2439 | 812 | 14 | 46634227-46639699 | 5.95 | 92.26 |
| *Malus domestica* | | | | | | | | |
| MdSUS1 | MDO.mRNA.g.10.40 | + | 1923 | 640 | 9 | Backbone_10:401891-404817 | 5.73 | 72.01 |
| MdSUS2 | MDO.mRNA.g.1121.1 | + | 1845 | 614 | 12 | Backbone_1121:11335-44391 | 5.99 | 70.16 |
| MdSUS3 | MDO.mRNA.g.1510.6 | - | 1113 | 370 | 7 | Backbone_1510:20583-22505 | 5.76 | 42.41 |
| MdSUS4 | MDO.mRNA.g.1510.9 | - | 1821 | 606 | 9 | Backbone_1510:35284-38090 | 5.65 | 68.79 |
| MdSUS5 | MDO.mRNA.g.1942.36 | + | 1677 | 558 | 10 | Backbone_1942:219828-223410 | 5.97 | 63.60 |
| MdSUS6 | MDO.mRNA.g.2408.15 | - | 1803 | 600 | 11 | Backbone_2408:100742-103798 | 6.24 | 67.96 |
| MdSUS7 | MDO.mRNA.g.2792.2 | + | 1899 | 632 | 15 | Backbone_2792:25664-30450 | 6.32 | 72.26 |
| MdSUS8 | MDO.mRNA.g.3274.4 | + | 1482 | 493 | 9 | Backbone_3274:36827-45555 | 5.73 | 56.03 |
| MdSUS9 | MDO.mRNA.g.3274.6 | + | 1404 | 467 | 8 | Backbone_3274:49131-52313 | 5.36 | 52.13 |
| MdSUS10 | MDO.mRNA.g.4524.3 | - | 1446 | 481 | 7 | Backbone_4524:19039-21275 | 6.62 | 54.59 |
| MdSUS11 | MDO.mRNA.g.693.6 | + | 2535 | 844 | 12 | Backbone_693 : 31554-35617 | 6.51 | 94.32 |
| MdSUS12 | MDO.mRNA.g.7.21 | - | 816 | 271 | 6 | Backbone_7 : 264058-265760 | 8.22 | 30.86 |
| *Oryza sativa* | | | | | | | | |
| OsSUS1 | LOC_Os02g58480 | + | 2538 | 845 | 11 | 35755136-35760862 | 5.99 | 96.15 |
| OsSUS2 | LOC_Os03g22120 | - | 2427 | 808 | 14 | 12674748-12680604 | 6.08 | 92.22 |
| OsSUS3 | LOC_Os03g28330 | - | 1701 | 566 | 10 | 16302728-16306057 | 5.68 | 64.23 |
| OsSUS4 | LOC_Os04g17650 | - | 2340 | 779 | 13 | 9662442-9667057 | 6.20 | 89.17 |
| OsSUS5 | LOC_Os04g24430 | + | 2565 | 854 | 16 | 14008320-14013239 | 7.77 | 97.81 |
| OsSUS6 | LOC_Os06g09450 | - | 2424 | 807 | 14 | 4796895-4801257 | 5.96 | 92.13 |
| OsSUS7 | LOC_Os07g42490 | + | 2448 | 815 | 14 | 25430598-25434904 | 6.15 | 93.11 |
| *Picea abies* | | | | | | | | |
| PaSUS1 | PAB00006776 | + | 2025 | 674 | 12 | MA_10427170 : 683-11170 | 6.00 | 77.32 |
| PaSUS2 | PAB00010305 | - | 2451 | 816 | 16 | MA_10430807:41869-51909 | 6.43 | 93.41 |
| PaSUS3 | PAB00011567 | - | 2601 | 866 | 17 | MA_10432094:2120-12852 | 6.32 | 99.51 |
| PaSUS4 | PAB00020688 | - | 1992 | 663 | 11 | MA_123269 : 2990-12025 | 5.89 | 75.20 |
| PaSUS5 | PAB00021357 | + | 2412 | 803 | 15 | MA_127762 : 1954-11249 | 5.61 | 91.06 |
| PaSUS6 | PAB00030334 | + | 777 | 258 | 0 | MA_1889 : 12939-13718 | 4.84 | 29.65 |
| PaSUS7 | PAB00035089 | - | 2031 | 676 | 14 | MA_27253 : 7418-18962 | 5.86 | 77.24 |
| *Physcomitrella patens* | | | | | | | | |
| PpSUS1 | Pp3c1_33460 | - | 2508 | 835 | 11 | 23598546-23602727 | 5.84 | 94.76 |
| PpSUS2 | Pp3c5_19770 | + | 2475 | 824 | 13 | 13945406-13951031 | 5.70 | 94.58 |
| PpSUS3 | Pp3c10_11330 | + | 2571 | 856 | 11 | 7611378-7615259 | 5.94 | 97.24 |
| PpSUS4 | Pp3c19_5850 | - | 2490 | 829 | 13 | 3270185-3275360 | 5.81 | 94.73 |
| *Populus trichocarpa* | | | | | | | | |
| PtSUS1 | Potri.002G202300 | + | 2433 | 810 | 14 | 16415973-16421156 | 5.90 | 92.94 |
| PtSUS2 | Potri.004G081300 | - | 2505 | 834 | 11 | 6705665-6709442 | 6.76 | 95.14 |
| PtSUS3 | Potri.006G136700 | + | 2409 | 802 | 12 | 11286612-11290388 | 6.17 | 92.14 |
| PtSUS4 | Potri.012G037200 | + | 2400 | 799 | 12 | 3315861-3319535 | 5.98 | 90.43 |
| PtSUS5 | Potri.015G029100 | + | 2430 | 809 | 12 | 2355838-2359496 | 6.48 | 91.77 |
| PtSUS6 | Potri.017G139100 | + | 2445 | 814 | 11 | 14732249-14736064 | 6.13 | 92.40 |
| PtSUS7 | Potri.018G063500 | - | 2415 | 804 | 12 | 7953808-7957574 | 6.23 | 92.49 |
| *Sorghum bicolor* | | | | | | | | |
| SbSUS1 | Sobic.001G344500 | + | 2448 | 815 | 13 | 63322534-63327118 | 6.03 | 92.96 |
| SbSUS2 | Sobic.001G378300 | + | 2427 | 808 | 14 | 66642863-66650032 | 6.37 | 92.29 |
| SbSUS3 | Sobic.004G357600 | + | 2589 | 862 | 10 | 68447180-68450780 | 8.35 | 97.70 |
| SbSUS4 | Sobic.010G072300 | - | 2406 | 801 | 14 | 5859487-5863836 | 5.82 | 91.71 |
| SbSUS5 | Sobic.010G276700 | - | 2589 | 862 | 15 | 60979397-60983598 | 7.81 | 102.48 |
| *Setaria italica* | | | | | | | | |
| SiSUS1 | Seita.1G378600 | + | 2577 | 858 | 11 | 41988015-41991623 | 6.96 | 97.81 |
| SiSUS2 | Seita.4G041500 | + | 2406 | 801 | 14 | 2944429-2948744 | 5.93 | 91.72 |
| SiSUS3 | Seita.4G288800 | - | 2568 | 855 | 15 | 40062014-40065954 | 7.17 | 97.60 |
| SiSUS4 | Seita.9G371700 | + | 2448 | 815 | 13 | 43203103-43207671 | 5.99 | 92.95 |
| SiSUS5 | Seita.9G410800 | + | 2427 | 808 | 13 | 46886703-46892013 | 6.10 | 92.34 |
| *Selaginella moellendorffii* | | | | | | | | |
| SmSUS1 | SMO203G0230 | + | 2517 | 838 | 14 | scaffold_40:543660-547057 | 6.11 | 95.44 |
| *Zea mays* | | | | | | | | |
| ZmSUS1 | Zm00001d014876 | - | 2580 | 859 | 15 | 5 : 66769897-66773844 | 8.19 | 98.02 |
| ZmSUS2 | Zm00001d029087 | - | 2550 | 849 | 13 | 1 : 57457862-57464526 | 6.24 | 96.44 |
| ZmSUS3 | Zm00001d029091 | - | 2205 | 734 | 13 | 1 : 57486293-57492974 | 6.38 | 83.50 |
| ZmSUS4 | Zm00001d045042 | + | 2358 | 785 | 14 | 9 : 10908224-10912666 | 6.19 | 89.91 |
| ZmSUS5 | Zm00001d047253 | - | 2451 | 816 | 13 | 9 : 124178734-124183200 | 6.03 | 92.93 |
| ZmSUS6 | Zm00001d051837 | - | 2550 | 849 | 10 | 4 : 171679816-171683167 | 7.63 | 96.27 |
| ZmSUS7 | Zm00001d053737 | - | 1434 | 477 | 6 | 4 : 240099038-240104000 | 6.44 | 53.28 |
