# Supplementary material for: Identification and analysis of sucrose synthase gene family associated with polysaccharide biosynthesis in Dendrobium catenatum by transcriptomic analysis
Source: PeerJ. 2022 Apr 5;10:e13222. doi: 10.7717/peerj.13222 (PMC8992646; doi:10.7717/peerj.13222)
Supplement: Table S11 [file peerj-10-13222-s016.doc]

**Table S11 The duplicated genes of SUS gene family in *D. catenatum* chromosomes.**

| gene name | **Gene**  gene model | gene name | **Syntenic regions**  gene model | **Type*** |
| --- | --- | --- | --- | --- |
| DcSUS1 | Dendrobium_GLEAN_10013680 | DcSUS9 | Dendrobium_GLEAN_10115731 | dispersed |
| DcSUS2 | Dendrobium_GLEAN_10032668 | DcSUS9 | Dendrobium_GLEAN_10115731 | dispersed |
| DcSUS3 | Dendrobium_GLEAN_10069518 | DcSUS2 | Dendrobium_GLEAN_10032668 | dispersed |
| DcSUS3 | Dendrobium_GLEAN_10069518 | DcSUS13 | Dendrobium_GLEAN_10069519 | tandem |
| DcSUS4 | Dendrobium_GLEAN_10069521 | DcTFIIF^#^ | Dendrobium_GLEAN_10076784 | dispersed |
| DcSUS5 | Dendrobium_GLEAN_10069526 | DcSUS11 | Dendrobium_GLEAN_10114912 | dispersed |
| DcSUS6 | Dendrobium_GLEAN_10089179 | DcSUS9 | Dendrobium_GLEAN_10115731 | dispersed |
| DcSUS7 | Dendrobium_GLEAN_10095005 | DcSUS12 | Dendrobium_GLEAN_10075975 | dispersed |
| DcSUS8 | Dendrobium_GLEAN_10105018 | DcSUS2 | Dendrobium_GLEAN_10032668 | dispersed |
| DcSUS9 | Dendrobium_GLEAN_10115731 | DcSUS10 | Dendrobium_GLEAN_10133317 | dispersed |
| DcSUS11 | Dendrobium_GLEAN_10114912 | DcSUS9 | Dendrobium_GLEAN_10115731 | dispersed |
| DcSUS12 | Dendrobium_GLEAN_10075975 | DcPPDT^#^ | Dendrobium_GLEAN_10076603 | dispersed |
| DcSUS13 | Dendrobium_GLEAN_10069519 | DcSUS2 | Dendrobium_GLEAN_10032668 | dispersed |
| DcSUS14 | Dendrobium_GLEAN_10105017 | DcSUS9 | Dendrobium_GLEAN_10115731 | dispersed |
| DcSUS14 | Dendrobium_GLEAN_10105017 | DcSUS8 | Dendrobium_GLEAN_10105018 | tandem |
| DcSUS15 | Dendrobium_GLEAN_10063746 | DcPPR^#^ | Dendrobium_GLEAN_10102652 | dispersed |

#: DcTFIIF: Transcription initiation factor IIF; DcPPDT: Pyridoxal phosphate-dependent transferase; DcPPR: Pentatricopeptide repeat.

*: dispersed: dispersed duplication (DSD); tandem: tandem duplication (TD).
